# *EGR1* regulates oxidative stress and aldosterone production in adrenal cells and aldosterone-producing adenomas

**DOI:** 10.1016/j.redox.2025.103498

**Published:** 2025-01-15

**Authors:** Yingxian Pang, Siyuan Gong, Martina Tetti, Zhuolun Sun, Sanas Mir-Bashiri, Martin Bidlingmaier, Thomas Knösel, Eckhard Wolf, Martin Reincke, Elisabeth Kemter, Tracy Ann Williams

**Affiliations:** a Medizinische Klinik und Poliklinik IV, Klinikum der Universität München, Ludwig-Maximilians-Universität München, Munich, Germany; b Institute of Pathology, Ludwig-Maximilians-Universität München, Munich, Germany; c Chair for Molecular Animal Breeding and Biotechnology, Gene Center and Department of Veterinary Sciences, Ludwig-Maximilians-Universität München, Munich, Germany

**Keywords:** Adrenal gland, Aldosterone-producing adenomas, Early growth response 1, Oxidative stress, CYP11B2, Hyperaldosteronism

## Abstract

Aldosterone-producing adenomas (APAs) are a major cause of primary aldosteronism, a common form of endocrine hypertension. Here, we demonstrate that Early Growth Response 1 (EGR1) plays a dual role in adrenal cell biology, regulating both oxidative stress and aldosterone production. Using RNA sequencing of RSL3-treated human adrenal cells and spatial transcriptomics of adrenal glands from patients with primary aldosteronism, we identify *EGR1* as a key gene associated with RSL3-related oxidative stress and APAs. We show that *EGR1* silencing decreases oxidative stress and increases *CYP11B2* gene expression and aldosterone production in adrenal cells, while its overexpression has the opposite effects. Notably, EGR1 expression is downregulated in APAs and aldosterone-producing micronodules compared to the adjacent adrenal cortex, which correlates in part with decreased levels of oxidative stress markers. The adrenal cortex of pigs with secondary hyperaldosteronism shows decreased immunostaining of EGR1 and a marker of oxidative stress, suggesting a potential link between EGR1 expression, oxidative stress levels, and adrenocortical function. These findings reveal a novel mechanism linking EGR1 to oxidative stress regulation and aldosterone production in adrenal cells, with potential implications for the pathogenesis of APAs and other adrenocortical tumors.

## Highlights

• EGR1 mediates RSL3-related oxidative stress in adrenocortical cells and adenomas.
• RSL3 inhibits CYP11B2 expression and aldosterone synthesis.
• Aldosterone-producing micronodules and adenomas show reduced EGR1 expression.
• *EGR1* knockdown decreases oxidative stress and enhances aldosterone production.
• EGR1 plays a dual role in promoting oxidative stress and aldosterone suppression in adrenal cells.

## Graphical abstract

**Figure:**
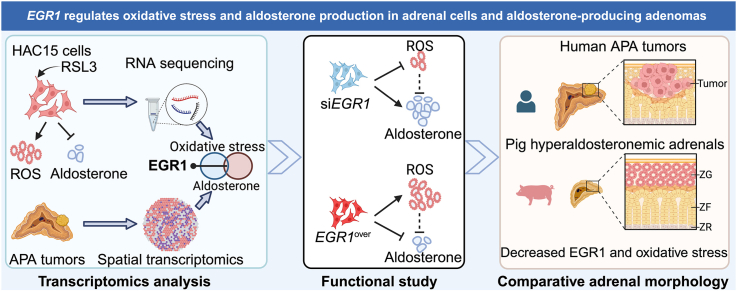

## 1 Introduction

Primary aldosteronism (PA) is a major cause of secondary hypertension, characterized by aldosterone overproduction independent of the renin-angiotensin-aldosterone system, leading to hypertension and an increased risk of adverse cardiovascular events. Aldosterone is produced by zona glomerulosa cells in the outer layer of the adrenal cortex, where CYP11B2 (aldosterone synthase) catalyzes the final steps in aldosterone biosynthesis. CYP11B2 immunostaining of adrenal gland sections allows visualization of potential aldosterone-secreting cells, helping identify the source of aldosterone excess in PA adrenals [[1], [2], [3]].

While CYP11B2 expression is typically regulated by angiotensin II (AngII) and potassium, PA can result from constitutive aldosterone production due to pathogenic variants in genes encoding ion channels (*KCNJ5* [4]*, CACNA1D* [5,6]*, CACNA1H* [7]*, CLCN2* [8,9]*, SLC30A1* [10]) or transporters (*ATP1A1* [6,11]*, ATP2B3* [11]). These variants ultimately increase intracellular calcium levels, stimulating *CYP11B2* gene transcription and aldosterone biosynthesis. Somatic variants in these PA-driver genes are found in aldosterone-producing adenomas (APAs) [[12], [13], [14]] and aldosterone-producing micronodules (APMs) [13], which are both major causes of PA [[15], [16], [17]].

Adrenocortical cells, particularly in the zona glomerulosa, are susceptible to oxidative stress due to their high steroidogenic activity, which generates significant reactive oxygen species (ROS) as a byproduct, primarily in mitochondria [18,19]. Despite robust antioxidant defenses, their high steroidogenic activity makes them vulnerable to redox imbalance [18,20] and the interplay between oxidative stress and adrenal steroidogenesis potentially contributes to adrenal disorders [18,21]. Recent research has also highlighted the sensitivity of adrenocortical cells to ferroptosis, an iron-dependent form of cell death mainly triggered by lipid peroxidation [22], thus emphasizing the importance of redox balance in adrenal physiology [19,[23], [24], [25]].

Oxidative stress can be assessed through various molecular markers reflecting different aspects of cellular redox status. Lipid peroxidation produces key markers such as malondialdehyde (MDA) and 4-hydroxynonenal (4-HNE) [26]. MDA results from ROS oxidation of polyunsaturated fatty acids in cell membranes, while 4-HNE can form adducts with proteins and DNA, potentially disrupting cellular functions. The process of lipid peroxidation is intricately linked to ferroptosis, as exemplified by RSL3 (RAS-selective lethal 3), a potent ferroptosis inducer that promotes lipid peroxidation through inhibition of the selenoprotein glutathione peroxidase 4 (GPX4) [27]. Cellular responses to oxidative stress can also be evaluated through the expression of specific proteins such as cyclooxygenase-2 (COX-2) [28] and transferrin receptor (TfR1) [29].

In this study, we investigated the relationship between oxidative stress and aldosterone production in adrenal cells and resected adrenal glands from patients with PA, aiming to elucidate the role of redox imbalance in the pathogenesis of this disease. Our previous research demonstrated elevated *GPX4* gene expression in APAs compared to adjacent zona glomerulosa cells, suggesting an adaptive response of tumor cells to oxidative stress [30]. Subsequent integrated spatial transcriptomics and spatial metabolomics analyses suggested that metabolic adaptation to cellular oxidative stress may contribute to the larger tumor size observed in APAs harboring a *KCNJ5* mutation [31]. These observations led us to hypothesize that oxidative stress may be a key factor in APA pathophysiology and growth. To investigate this further, we conducted RNA sequencing on RSL3-treated human adrenocortical cells and compared the results with spatial transcriptomics datasets we previously generated from adrenal cryosections containing both APA and adjacent cortical tissue [31]. We then identified common differentially expressed genes (DEGs) between these two datasets, which highlighted *EGR1* as a key molecular player in both the adrenal oxidative stress response and APA pathogenesis. We determined the functional significance of *EGR1* through targeted *in vitro* assays in human adrenocortical cells. Furthermore, we characterized the spatial distribution of oxidative stress markers in human adrenal specimens surgically removed from patients with an APA and in adrenal sections from a pig model of secondary hyperaldosteronism. We demonstrate that the transcription factor EGR1 plays a dual function in the adrenal cortex by modulating the cellular response to oxidative stress and suppressing aldosterone production. Our findings reveal a novel role for EGR1 in redox mechanisms of aldosterone production under physiological and pathophysiological conditions.

## 2 Results

### 2.1 Human adrenal cell ferroptosis and aldosterone production

**Fig. 1:**
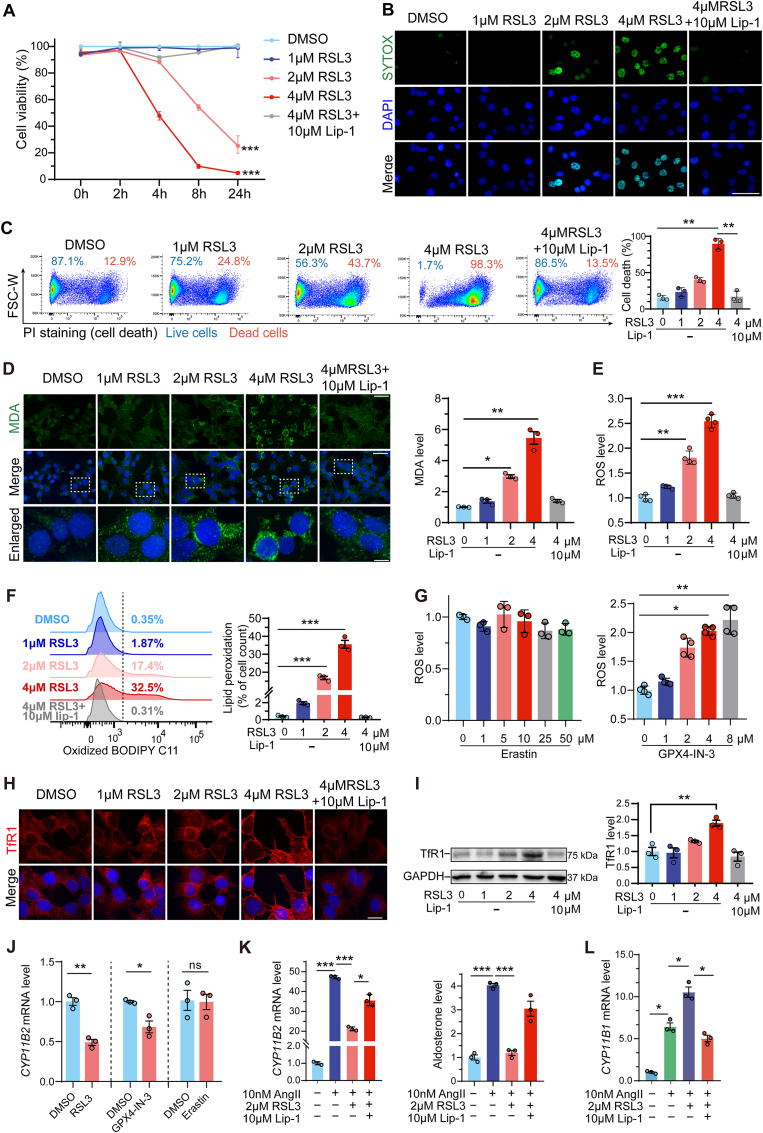
RSL3-induced oxidative stress downregulates aldosterone synthase gene expression and aldosterone production in human adrenocortical cells. A. HAC15 cell viability after treatment with RSL3 (0–4 μM) or 10 μM Lip-1+4 μM RLS3 for 0–24 h (n = 3 independent experiments). **B and C.** Cell death analysis of HAC15 cells treated with RSL3 (0–4 μM) or 10 μM Lip-1+4 μM RLS3 for 4 h by cytoimmunofluorescence and flow cytometry. Representative figures are shown, n = 3 independent experiments. Scale bars = 25 μm. **D and E.** MDA cytoimmunofluorescence (n = 3) and quantification and ROS assay (n = 4) of HAC15 cells treated with RSL3 (0–4 μM) or 10 μM Lip-1+4 μM RLS3 for 4 h. Scale bars, 20 μm (upper), 5 μm (lower). **F.** Representative flow cytometry analysis images and quantification of BODIPY C11-positive cells (lipid peroxidation; n = 3 independent experiments) after treatment with RSL3 (0–4 μM) or 10 μM Lip-1+4 μM RLS3 for 4 h. **G.** ROS assay of HAC15 cells treated with 0–50 μM Erastin or 0–8 μM GPX4-IN-3**. H and I.** TfR1 cytoimmunofluorescence and TfR1 Western blot analysis(n = 3) in HAC15 cells treated with RSL3 (0–4 μM) or 10 μM Lip-1+4 μM RLS3 for 4 h. Scale bars = 10 μm. **J.***CYP11B2* (aldosterone synthase) gene expression analysis of HAC15 cells treated with 2 μM RSL3 or 2 μM GPX4-IN-3 for 6 h, or 25 μM Erastin for 24 h (n = 3 independent experiments). **K and L.***CYP11B2* and *CYP11B1* (11β-hydroxylase) gene expression analysis and quantification of secreted aldosterone from HAC15 cells treated with 10 nM AngII, 10 nM AngII+2 μM RLS3 or 10 nM AngII+2 μM RLS3+10 μM Lip-1 for 14 h (n = 3 independent experiments). Data were presented as the mean ± standard error of mean. ∗*P* < 0.05, ∗∗*P* < 0.01, ∗∗∗*P* < 0.001.

HAC15 cells were treated with different concentrations of RSL3, an inducer of cellular oxidative stress, in the presence or absence of liproxstatin-1 (Lip-1), an antioxidant inhibitor of lipid peroxidation. RSL3 induced a significant dose-dependent decrease in HAC15 cell viability (Fig. 1A) and increase in cell death (Fig. 1B and C), both of which were inhibited by Lip-1. Additionally, RSL3 treatment significantly elevated Lip-1-sensitive cellular MDA, ROS and lipid peroxidation levels in HAC15 cells (Fig. 1D–F). The selective GPX4 inhibitor GPX4-IN-3 caused a dose-dependent increase in ROS levels in HAC15 cells [32]. In contrast, treatment with Erastin at concentrations up to 50 μM had no effect on ROS production in HAC15 cells (Fig. 1G). TfR1 expression was also markedly increased in response to 4 μM RSL3 (Fig. 1H and I). These results indicate that the cell death induced by RSL3 in HAC15 cells is consistent with ferroptosis, a form of regulated cell death primarily driven by lipid peroxidation.

To evaluate the effect of oxidative stress on aldosterone secretion in HAC15 cells, we treated the cells with RSL3, GPX4-IN-3, Erastin, or combinations of AngII, RSL3, and Lip-1. Oxidative stress induced by RSL3 or GPX4-IN-3 resulted in a decrease in *CYP11B2* expression, whereas Erastin had no effect (Fig. 1J). Cells were treated with 10 nM AngII to stimulate aldosterone production, with or without 2 μM RSL3 to induce oxidative stress in the presence or absence of the antioxidant Lip-1 (10 μM). AngII (10 nM) significantly increased both *CYP11B2* gene expression and aldosterone secretion (Fig. 1K and L). This stimulatory effect was markedly inhibited by 2 μM RSL3 whilst Lip-1 reversed the inhibitory effect of RSL3 on AngII-induced *CYP11B2* expression and aldosterone secretion. Additionally, RSL3 showed a stimulatory effect on the expression of the homologous *CYP11B1* gene (Fig. 1L).

### 2.2 Identification of oxidative stress-related genes in adrenal cells

**Fig. 2:**
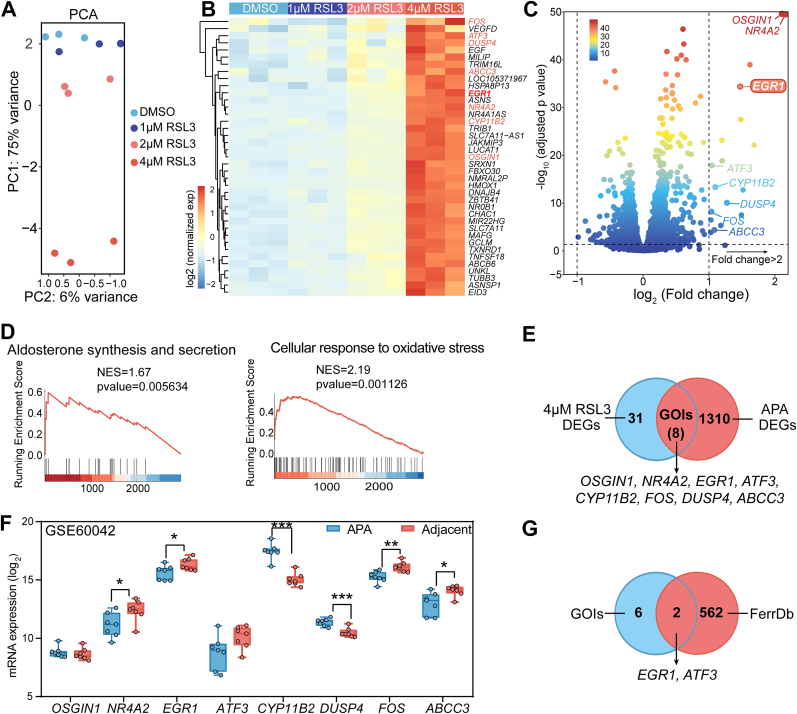
Identification and validation of oxidative stress-related genes in oxidative stress-induced human adrenocortical cells. **A.** Principal component analysis (PCA) of RNA-sequencing data of HAC15 cells treated with RSL3 (0–4 μM) for 3 h (three biologically independent samples for each condition). **B.** Heatmap showing the differentially expressed genes (DEGs) of RNA-sequencing. **C.** Volcano plot highlighting genes of interest (GOIs) in HAC15 cells induced by 4 μM RLS3 for 3 h compared to DMSO. The color scale bar indicates log_10_ (adjusted p value). **D,** Related pathways in GSEA enrichment analysis in HAC15 cells treated with 4 μM RSL3. **E,** Venn diagram highlighting overlapping DEGs of interest induced by 4 μM RLS3 versus DMSO vehicle-only control (RNA sequencing) and in APA versus paired adjacent cortex (spatial transcriptomics). **F,** Expression of genes of interest in APA and adjacent adrenal cortex from GEO dataset GSE60042 (7 patients). **G,** Venn diagram showing common genes between the GOIs shown in panel F and ferroptotic genes from the FerrDb database. ∗*P* < 0.05, ∗∗*P* < 0.01, ∗∗∗*P* < 0.001.

Genes associated with cellular oxidative stress in adrenal cells were identified through RNA sequencing of RSL3-treated HAC15 cells (Fig. 2A), followed by comprehensive bioinformatics analysis. We identified 39 upregulated DEGs in 4 μM RSL3-treated HAC15 cells compared to the DMSO-treated control group (Fig. 2B and C). Gene Set Enrichment Analysis (GSEA) indicated that these genes were related to aldosterone synthesis and secretion, as well as oxidative stress responses (Fig. 2D).

Analysis of spatial transcriptome data from six PA adrenal glands revealed that eight of the 39 DEGs identified from RSL3-treated HAC15 cells also exhibited differential expression in APAs compared to the adjacent adrenal cortex (Fig. 2E). To validate the expression of these eight genes of interest in APAs, we analyzed a Gene Expression Omnibus (GEO) dataset (GSE60042) [33], which showed that *NR4A2*, *EGR1*, *FOS*, and *ABCC3* gene expression was downregulated in APA tumor tissue compared to the adjacent cortex, whereas *CYP11B2* and *DUSP4* were upregulated (Fig. 2F). Of the eight genes of interest, *EGR1* and *ATF3* are listed in the FerrDb database (Fig. 2G), a comprehensive resource for ferroptosis regulators. This suggests their potential role in lipid peroxidation-mediated oxidative stress processes that contribute to the pathophysiology of APAs. Since *ATF3* expression was very low in both APA tumor and adjacent tissues from the spatial transcriptome analysis, we selected *EGR1* for further investigation.

### 2.3 Differential expression of EGR1 in response to oxidative stress and in APAs

**Fig. 3:**
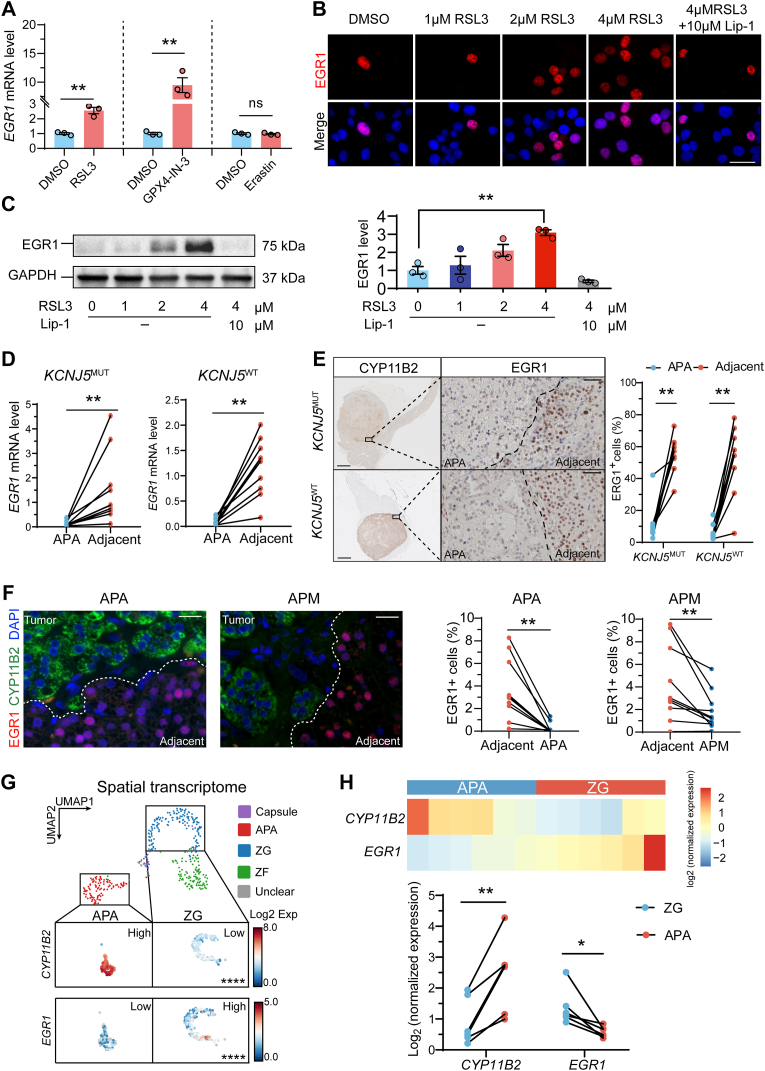
EGR1 expression in oxidative stress-induced adrenocortical cells and aldosterone-producing adenomas versus adjacent adrenal cortex. **A.** Gene expression analysis of *EGR1* in HAC15 cells treated with 4 μM RSL3 or 2 μM GPX4-IN-3 for 4 h or 25 μM Erastin for 24 h (n = 3 independent experiments). **B and C.** Cytoimmunofluorescence and Western blot and gene expression analysis of EGR1 expression in HAC15 cells treated with RSL3 (0–4 μM) or 10 μM Lip-1+4 μM RLS3 for 4 h. Representative figures are shown, n = 3 independent experiments. Scale bars = 20 μm. **D.** Gene expression analysis of *EGR1* in APA and adjacent cortex of 10 patients with and 10 without *KCNJ5* mutated APAs. **E.** CYP11B2 (aldosterone synthase) and EGR1 immunohistochemistry was performed on 18 adrenal glands, comprising nine with *KCNJ5*-mutated APAs and nine with wild-type *KCNJ5* APAs and EGR1-immunopositive cells were quantified. Representative immunostainings are shown (left) and the proportion of EGR1-positive cells is also shown (right). Scale bars, 200 μm (left), 50 μm (right). **F.** Representative CYP11B2-EGR1 double-immunofluorescence of an APA and an APM, with quantification in nine APAs and nine APMs. Scale bar = 10 μm. **G.** Spatial transcriptome data showing inverse *CYP11B2* and *EGR1* gene expression profiles in an adrenal sample with an APA. **H.** Integrated *CYP11B2* and *EGR1* gene expression profiles from spatial transcriptome data of six adrenal samples with an APA and attached adjacent adrenal cortex in the same captured area. ∗*P* < 0.05, ∗∗*P* < 0.01.

To investigate the effect of oxidative stress on EGR1 expression, HAC15 cells were treated with RSL3, GPX4-IN-3 or Erastin. *EGR1* gene expression was significantly increased by 4 μM RSL3 or 2 μM GPX4-IN-3 compared to controls. However, treatment with 25 μM Erastin did not affect *EGR1* gene expression levels relative to controls (Fig. 3A). RSL3 led to a dose-dependent increase in EGR1 protein expression, as demonstrated by both immunofluorescence (Fig. 3B) and Western blotting (Fig. 3C), an effect that was reversed by 10 μM Lip-1.

We then investigated EGR1 gene and protein expression in surgically removed adrenal glands from patients with PA. *EGR1* mRNA levels were significantly downregulated in 20 APA tumor specimens compared with their paired adjacent adrenal cortex. This observation was independent of the presence of a *KCNJ5* pathogenic variant (Fig. 3D). Immunofluorescence and immunohistochemical staining of adrenal sections confirmed the decreased expression of EGR1 in 18 APAs compared to the adjacent cortex (nine with a *KCNJ5* pathogenic variant and nine with wild-type *KCNJ5*) (Fig. 3E and F). Decreased EGR1 expression was also observed in nine APMs compared to the adjacent CYP11B2-negative zona glomerulosa cells (Fig. 3F). Finally, spatial transcriptome analysis of adrenal cryosections also demonstrated downregulation of *EGR1* gene expression in APA tumor regions compared to the adjacent zona glomerulosa in six PA adrenal samples (Fig. 3G and H).

### 2.4 EGR1 modulates oxidative stress responses and CYP11B2 expression in adrenal cells

**Fig. 4:**
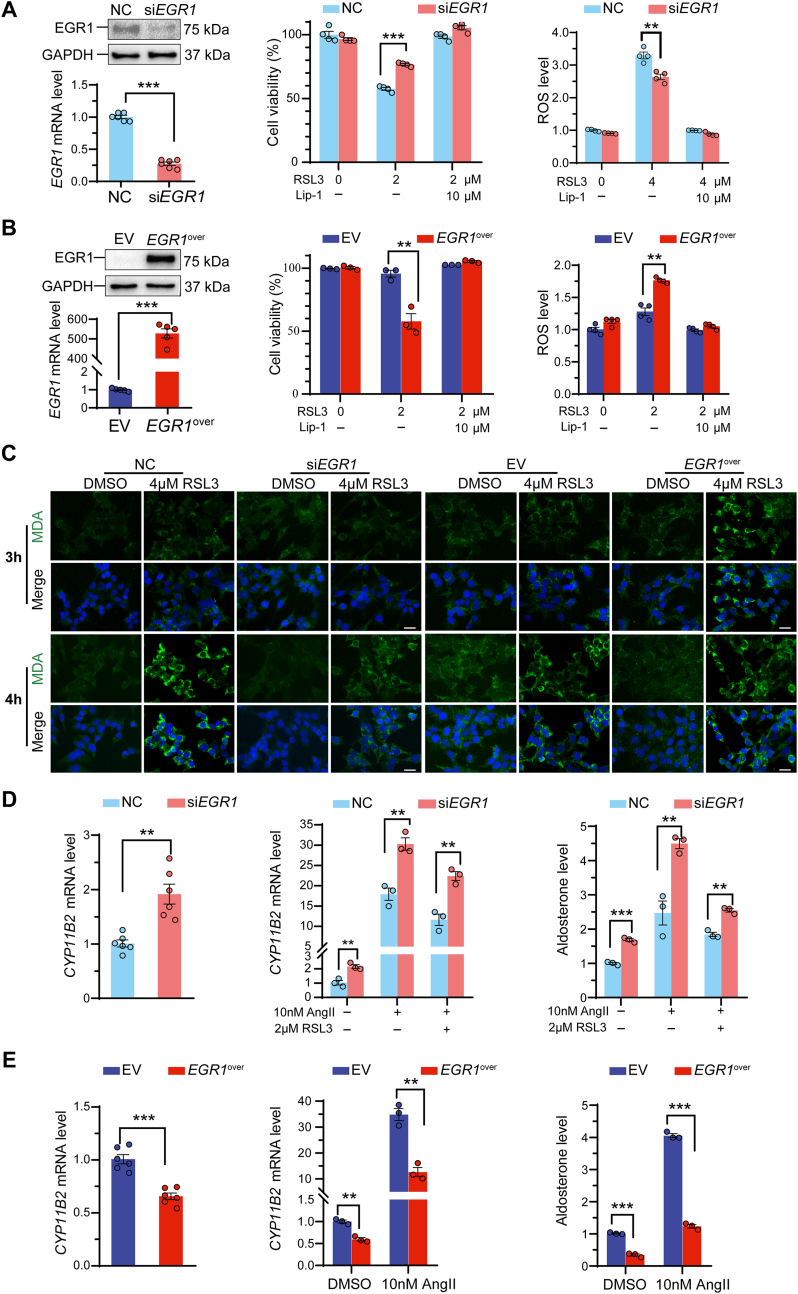
*EGR1* promotes oxidative stress and inhibits aldosterone production in human adrenocortical cells. **A and B.** Gene expression (n = 6 independent experiments) and Western blot analysis (n = 3) of EGR1 in HAC15 cells after *EGR1* gene silencing or overexpression. NC, negative control. EV, empty vector. Cell viability in HAC15 cells after *EGR1* silencing (**A**, n = 4) and *EGR1*-overexpression (**B**, n = 3) treated with 2 μM RSL3 or 10 μM Lip-1+2 μM RLS3 for 4 h. ROS level in the *EGR1*-silenced and *EGR1*-overexpressing HAC15 cells treated with RSL3 or Lip-1+ RLS3 for 4 h (**A, B,** n = 4 independent experiments). **C,** Representative MDA staining in the HAC15 *EGR1*-silenced HAC15 cells treated with 4 μM RSL3 for three and 4 h, and *EGR1*-overexpressing HAC15 cells treated with 4 μM RSL3 for three and 4 h. Scale bars = 25 μm. **D and E.** Gene expression analysis of *CYP11B2* after *EGR1* silencing or overexpression in HAC15 cells (n = 6 independent experiments). *CYP11B2* gene expression and aldosterone production after *EGR1* silencing of HAC15 cells treated with 10 nM AngII, 2 μM RSL3 or 10 nM AngII+2 μM RSL3 for 12 h (**D**, n = 3 independent experiments). *CYP11B2* gene expression and aldosterone production in *EGR1* overexpressing HAC15 cells treated with and without 10 nM AngII for 12 h (**E**, n = 3 independent experiments). Data were presented as the mean ± standard error of mean. ∗∗*P* < 0.01, ∗∗∗*P* < 0.001.

To elucidate the role of EGR1 in HAC15 cells, we generated cells with either silenced or overexpressed *EGR1* (Fig. 4A and B). *EGR1*-silenced HAC15 cells showed increased cell viability compared to control HAC15 cells, effects that were reversed by Lip-1. In contrast, *EGR1*-overexpressing cells exhibited the opposite response. Furthermore, assays of ROS levels and immunofluorescence imaging of MDA demonstrated lower levels of both markers in *EGR1*-silenced HAC15 cells, but higher production in *EGR1*-overexpressing cells compared to controls (Fig. 4A–C).

The expression of *CYP11B2* was upregulated in *EGR1*-silenced HAC15 cells, while it was downregulated in *EGR1*-overexpressing cells (Fig. 4D and E). Stimulation of HAC15 cells with 10 nM AngII increased *CYP11B2* gene expression as expected, but this increase was higher in *EGR1*-silenced cells compared to controls. When co-treated with 10 nM AngII and RSL3, the increase in *CYP11B2* gene expression in *EGR1*-silenced HAC15 cells was less pronounced compared to control cells. Furthermore, aldosterone secretion was significantly increased in *EGR1*-silenced HAC15 cells, and this difference was attenuated by RSL3-induced oxidative stress (Fig. 4D). Consistently, in *EGR1*-overexpressing cells, the basal and AngII-stimulated *CYP11B2* gene expression and aldosterone levels were both lower compared to controls (Fig. 4E).

### 2.5 Oxidative stress markers in adrenocortical cells and hyperaldosteronism adrenal glands

**Fig. 5:**
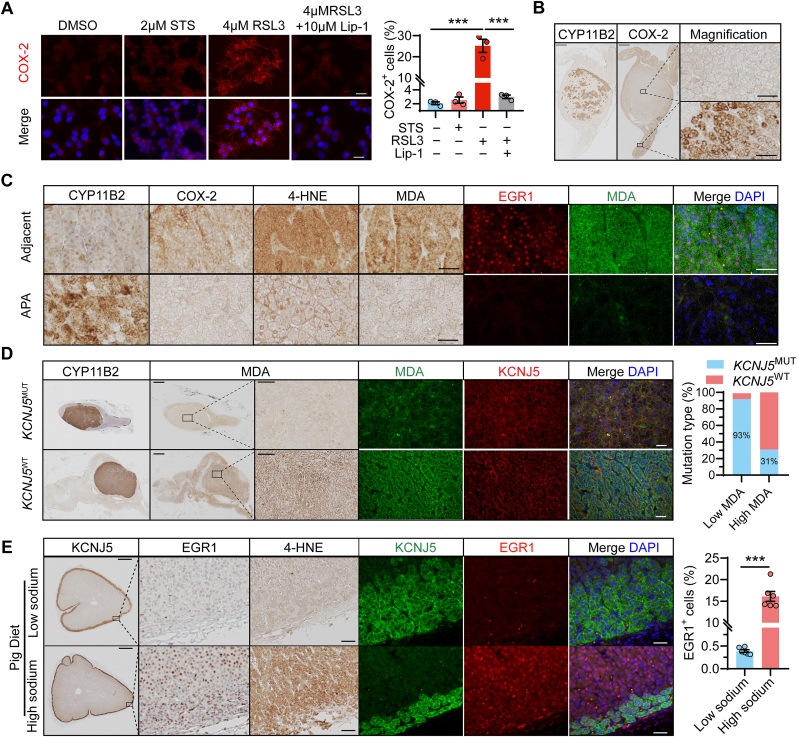
Suppression of oxidative stress under conditions of hyperaldosteronism. **A.** Immunofluorescence and quantification showing increased expression of the oxidative stress marker COX-2 in HAC15 cells treated with the ferroptosis inducer RSL3 but not with an inducer of apoptosis staurosporine (STS) (n = 3 independent experiments). **B.** CYP11B2 and COX-2 immunohistochemistry showing reduced COX-2 immunostaining in the APA compared to the adjacent cortex. Scale bars, 2 mm (upper left), 50 μm (lower right). **C.** CYP11B2, COX-2, 4-HNE and MDA immunohistochemistry and EGR1 and MDA immunofluorescence of an APA and its adjacent cortex. The immunostainings are from the same adrenal sample. Scale bars, 50 μm. **D.** MDA immunostaining in patients with APA. Scale bars, 2 mm (upper left), 200 μm (upper right), 50 μm (lower right). **E.** KCNJ5, EGR1, and 4-HNE immunohistochemistry and KCNJ5-EGR1 double immunofluorescence of adrenal glands from pigs fed a low or high sodium diet. Quantification of EGR1 expression was performed in two regions from each pig adrenal gland. KCNJ5 is a marker of aldosterone-producing outer zona glomerulosa layer in pig adrenal glands. Sodium restriction activates the renin-angiotensin-aldosterone system and stimulates expansion of the adrenal zona glomerulosa layer and aldosterone production. Immunostaining of EGR1 and the oxidative stress marker 4-HNE are markedly reduced in adrenals of pigs with hyperaldosteronism caused by low sodium compared to a high sodium diet. Scale bars, 1 mm (upper), 50 μm (lower). Representative figures are shown.

To further investigate the relationship between oxidative stress and aldosterone production, we assessed several markers of oxidative stress *in vitro* and *in vivo*. COX-2 showed increased immunofluorescence in HAC15 cells treated with RSL3 (Fig. 5A) and immunohistochemistry revealed notably lower COX-2 expression in APAs compared to adjacent adrenal tissue (Fig. 5B). Further, immunohistochemistry of 4-HNE and MDA, and double immunofluorescence labelling of EGR1 and MDA, demonstrated decreased levels of these markers and EGR1 in APA tumor tissues relative to the adjacent cortex (Fig. 5C). APAs exhibited reduced MDA levels compared to the adjacent adrenal cortex in 14 of 40 (35 %) adrenal tissues, while 20 (50 %) displayed comparable MDA levels, and 6 (15 %) showed elevated levels (Fig. 5D). APAs with lower MDA levels relative to the adjacent adrenal cortex more frequently carried a *KCNJ5* mutation compared to those with MDA levels that were comparable to or higher than the adjacent adrenal tissue (93 % vs. 31 %, *P* < 0.001). This observation suggests an inverse relationship between oxidative stress and aldosterone production. To explore this further, we utilized a pig model of secondary hyperaldosteronism induced by dietary salt restriction, which activates the renin-angiotensin-aldosterone system [34]. We compared adrenals of pigs fed a low-sodium diet (stimulating aldosterone production) to those of pigs fed a high-sodium diet (suppressing aldosterone production). Expression levels of EGR1 and the oxidative stress marker 4-HNE were lower throughout the adrenal cortex of pigs on the low-sodium diet compared to those on the high-sodium diet (Fig. 5E).

## 3 Discussion

Our findings reveal a previously unrecognized dual regulatory role of EGR1 in adrenal function, demonstrating for the first time that it modulates both the cellular response to oxidative stress and aldosterone production in adrenocortical cells. The inverse relationship between oxidative stress markers and aldosterone production, observed across multiple modalities including human adrenocortical cells, a pig model of secondary hyperaldosteronism, and human APAs, provides novel insights into the complex regulation of adrenal homeostasis. These results advance our understanding of adrenal physiology and open new avenues for investigating the pathophysiology of APAs and other adrenal disorders characterized by dysregulated steroidogenesis.

We report that RSL3-mediated oxidative stress in human adrenal cells *in vitro* induces EGR1 expression while inhibiting *CYP11B2* transcription and aldosterone production. We further validated the regulatory role of *EGR1* on *CYP11B2* expression and aldosterone synthesis by *EGR1* overexpression or silencing, which yielded consistent results. Previous studies have shown that *EGR1* decreases *CYP11B2* promoter activity and both basal and angiotensin II-stimulated aldosterone secretion [35,36]. Our results confirm and extend these findings by providing a mechanistic link through oxidative stress. Notably, EGR1 gene and protein expression was decreased in both APAs and APMs compared to adjacent adrenocortical cells. This reduction aligned with the adrenal distribution pattern of oxidative stress markers and was inversely correlated with the CYP11B2 expression profile, supporting a role for EGR1 in the pathophysiology of PA. In our pig model of secondary hyperaldosteronism induced by a low sodium diet, EGR1 immunostaining was markedly reduced in the aldosterone-producing zona glomerulosa layer compared to the rest of the adrenal cortex thereby supporting the negative regulatory role of EGR1 on aldosterone production. This suggests that EGR1 agonists could inhibit aldosterone secretion and putatively offer a novel therapeutic strategy for the treatment of APAs in individuals in whom surgery is undesired. Troglitazone selectively induces *EGR1* gene expression and has antitumor activity [37]. However, its hepatotoxicity excludes its therapeutic use [38] and there is currently no clinical evidence supporting the use of EGR1 agonists as a treatment for PA.

Emerging evidence suggests EGR1 modulates redox states and oxidative stress in various pathological conditions [39,40]. Our study extends this to adrenocortical cells in which *EGR1* overexpression enhanced the response to RSL3, as shown by increased production of ROS and the lipid peroxidation marker MDA. Although RSL3 was long considered a direct inhibitor of GPX4 [27], recent biochemical assays suggest another selenoprotein, TXNRD1 (thioredoxin reductase 1), as a primary RSL3 target [41]. TXNRD1 inhibition disrupts cellular redox homeostasis, leading to lipid peroxide accumulation and oxidative events similar to those observed with direct GPX4 inhibition by GPX4-IN-3 [32]. This oxidative stress is further amplified by EGR1-mediated transcriptional regulation of genes involved in lipid metabolism and antioxidant defense, such as *NR4A3*, *ALOX5*, *HMOX1* and *GPX4* [[40], [41], [42], [43], [44]].

In this study, RSL3 and GPX4-IN-3 induced dose-dependent increases in ROS levels, *EGR1* expression, and decreases in *CYP11B2* gene expression in human adrenal cells. Surprisingly, Erastin did not elicit these effects. While Erastin (0.1–10 μM) typically elevates ROS within 24 h in various cell lines [45,46], HAC15 cells treated with up to 50 μM Erastin showed no changes in ROS production, *EGR1*, or *CYP11B2* gene expression. Erastin inhibits the cystine/glutamate antiporter system (system x_c_−), indirectly affecting GPX4 by depleting intracellular glutathione. The differential response between RSL3/GPX4-IN-3 and Erastin in human adrenal cells suggests that direct inhibition of GPX4 may be more critical for oxidative stress induction in these cells than indirect inhibition via system x_c_−. This heightened sensitivity to direct GPX4 inhibition could be attributed to the high steroidogenic activity and lipid metabolism characteristic of adrenal cells, which may render them particularly vulnerable to disruptions in antioxidant defense mechanisms.

Additionally, our study indicates that *NR4A2 (NURR1)* may play a role in oxidative stress regulation, as evidenced by its elevated expression under RSL3-induced oxidative stress conditions in this study. Many studies have linked *NR4A2* to cell cycle regulation, cellular senescence, and oxidative stress [47,48]. In the context of PA, *NR4A2* functions as a transcriptional regulator of *CYP11B2* expression [49]. However, its specific role in mediating oxidative stress in PA remains to be fully elucidated and warrants further experimental investigation.

Our findings reveal an apparent paradox: while oxidative stress within the adrenal gland can suppress aldosterone production, excessive aldosterone secretion may conversely contribute to oxidative stress-mediated cardiac and renal damage [49]. This bidirectional relationship highlights the complex interactions between aldosterone and oxidative stress in the pathophysiology of PA and its associated complications.

EGR1 acts directly on the *CYP11B2* promoter [35] and likely modulates *CYP11B2* expression and aldosterone synthesis through oxidative stress-mediated pathways. Our experiments with the antioxidant Lip-1 support this hypothesis. Lip-1 effectively reversed both the inhibitory effect of RSL3 on *CYP11B2* expression and aldosterone secretion, as well as its stimulatory effect on *EGR1* expression in human adrenal cells. Additionally, we noted low levels of both EGR1 and oxidative stress markers in APA tumors. This suggests that APA cells may have adapted to mitigate excessive oxidative damage, possibly through enhanced antioxidant defenses or metabolic reprogramming [30,31,50]. Such adaptations parallel observations in cancer research, where tumors can develop mechanisms to manage oxidative stress [51].

Markers related to iron intake/efflux and glutathione metabolism warrant further investigation in APAs, as they might provide insights into APA pathophysiology and oxidative stress mechanisms. These oxidative stress pathways appear to be closely linked to EGR1 function in adrenal cells. While several studies have shown that EGR1 can promote cell and tumor proliferation [[52], [53], [54]], our findings suggest a different role in the context of PA. Specifically, EGR1 is more likely to inhibit *CYP11B2* expression and aldosterone secretion by regulating oxidative stress rather than directly affecting cell proliferation.

In conclusion, this study integrates data from *in vitro* and spatial transcriptomics, functional gene characterization, a pig model of hyperaldosteronism and surgical adrenal gland specimens to elucidate the regulatory role of EGR1 in oxidative stress and aldosterone production under physiological and pathophysiological conditions. The EGR1-oxidative stress-aldosterone axis described here offers new perspectives on cellular adaptation in endocrine tumors and highlights potential intervention targets in APAs involved in redox regulation and lipid metabolism.

## 4 Materials and methods

### 4.1 Cell culture

Human adrenocortical (HAC15) cells were cultured in Dulbecco's Modified Eagle's Medium/Nutrient Mixture F-12 (DMEM/F12, Gibco, 11330-032) with supplements of 10 % Cosmic Calf serum (CCS, HyClone, SH30087.03), 1 % insulin-transferrin-selenium-sodium pyruvate (ITS, Gibco, 51300-44), 1 % antibiotic-antimycotic (Gibco, 15240-062), and 0.01 % Gentamicin (Gibco, 15750-060). The cells were maintained at 37 °C under an atmosphere of 95 % H2O and 5 % CO2. For experimental treatments, cells were cultured in a starvation medium (DMEM/F12 containing 0.1 % CCS) both before and after the addition of stimulants.

### 4.2 Large animal model of secondary hyperaldosteronism

Formalin-fixed paraffin-embedded (FFPE) adrenal glands from 6-week-old male German Landrace DanBred pigs fed a 14-day 0.04 % sodium diet (low sodium, n = 3) to activate the renin-angiotensin-aldosterone system or a 0.7 % sodium (high sodium, n = 3) were from a previous study [34].

### 4.3 Patients and tissue samples

APAs were surgically removed from patients diagnosed with unilateral PA at the Klinikum der Ludwig-Maximilians-Universität München, Munich, Germany. The presence of an APA in the resected adrenal tissue was confirmed by CYP11B2 immunohistochemistry [1]. *KCNJ5* mutations in APAs were identified following a previously established protocol [24]. Spatial transcriptomics data were from our previous study (GSE274314) [24]. The study was conducted in accordance with local ethics committee guidelines and approved by the institutional review board (project number 379-10 and 24–0696). All patients provided written informed consent before their samples were included in the study.

### 4.4 Next generation sequencing and bioinformatics analysis

Total RNA was extracted from HAC15 cells treated with 0–4 μM RSL3 for 3 h, and reverse transcribed according to the manufacturer's instructions. mRNA sequencing was conducted by Eurofins Genomics (Germany) using Illumina paired-end sequencing with a read length of 2x150 bp, guaranteeing 30 million read pairs (±3%). Differentially expressed genes were identified using the DESeq2 package in R software and visualized using heatmap and volcano plots. Significant differential expression was defined by an adjusted *P* value < 0.05 and an absolute value of fold change >2. Gene set enrichment analysis (GSEA) was conducted using the ClusterProfiler package to identify enriched pathways. Kyoto Encyclopedia of Genes and Genomes (KEGG) pathway enrichment was performed using the online bioinformatics tool Sangerbox (http://www.sangerbox.com/tool). Ferroptosis-related genes were downloaded from FerrDb database (http://www.zhounan.org/ferrdb/). The differential expression of genes of interest was validated by analyzing transcriptome data comparing APAs with adjacent adrenal cortex, obtained from the GEO database (accession no. GSE60042) [33].

### 4.5 Generation of adrenal cells with gene-specific knockdown or overexpression

HAC15 cells (1.5 × 10^6^ cells/well) were transfected with either 2 μL of a 100 μM *EGR1* siRNA (Invitrogen, 4390824) or 3 μg *EGR1* cDNA (Origene, RC209956) using the Cell Line Nucleofector™ Kit R (Amaxa, VCA-1001) and the Nucleofector™ 2b device (Lonza, program X-005) according to the manufacturer's instructions. Transfected cells were harvested for real-time qPCR analysis after 48 h or Western blot analysis after 72 h to measure levels of gene or protein expression, respectively.

### 4.6 Cell viability assay

The effect of RSL3 on HAC15 cell viability was assessed using the water-soluble tetrazolium salt-1 (WST-1, Roche, 11644807001) assay. Briefly, HAC15 cells (3.2 × 10^4^ cells/well) or transfected (2.0 × 10^4^ cells/well) cells were seeded on 96-well plates in starvation medium and incubated for 24 h before treatment with 0–4 μM [1S, 3R]-RSL3 (RSL3, TOCRIS, 6118) or 10 μM Lip-1 (TOCRIS, 6113) + 4 μM RSL3 for 4 h. After incubation with WST-1 solution at 37 °C and 5 % CO_2_ for 3 h, absorbance was measured at 450 nm and 690 nm using a FLUOstar Omega plate reader (BMG LABTECH).

### 4.7 Cell death measurement and lipid peroxidation assay

HAC15 cells (7.5 × 10^5^ cells/well) were seeded into 6-well plates, and incubated for 24 h, followed by a further 24 h in starvation medium. Cells were then treated with 0–4 μM RSL3 or 10 μM Lip-1 + 4 μM RSL3 for 4 h. Subsequently, all cells were harvested and stained with propidium iodide (PI) (eBioscience, 00-6990-50) or SYTOX™ Green Nucleic Acid Stain (Invitrogen™, S7020) to detect cell death, or BODIPY 581/591C11 (Invitrogen™, D3861) to measure lipid peroxidation. Fluorescence intensity was measured using an LSRFortessa™ cell analyzer (BD Bioscience, 647794) on PE-Texas Red or FITC channel. All experiments were performed in triplicate, with a minimum of 30,000 single cells acquired per sample and analyzed using FlowJo software (version 10.8.1).

### 4.8 ROS assay

ROS levels in HAC15 cells treated with RSL3, GPX4-IN-3 (MedChemExpress, HY-141809) or Erastin (TOCRIS, 5449) were assessed using the DCFDA/H2DCFDA kit (Abcam, ab113851) following the manufacturer's protocol.

### 4.9 Real-time qPCR analysis

Total RNA was extracted using the Maxwell RSC simplyRNA tissue kit (Promega, AS1340). Reverse transcription was performed on 500 ng of RNA (GoScript™ reverse transcriptase kit, Promega, A2791) according to the manufacturer's protocol. Universal Probes Supermix (Bio-rad, 1725131) was used to perform real-time qPCR on the QuantStudio 5 with the following TaqMan probes: *EGR1* (Thermofisher Scientific, Hs00152928_m1), *CYP11B2* (Thermofisher Scientific, Hs01597732_m1), *CYP11B1* (Thermofisher Scientific, Hs01596406_m1) and *GAPDH* (Thermofisher Scientific, Hs02786624_g1). The 2^-ΔΔCt^ method was used to quantify gene expression using *GAPDH* as the endogenous reference gene.

### 4.10 Western blot analysis

Western blotting was performed as previously described [24]. The primary antibodies were: EGR1 (1:1000, Cell Signaling Technology, 4153), TfR1/CD71 (clone 3F3-FMA, 1:1000, Sigma-Aldrich, MABC1765), or GAPDH (1:2500, Cell Signaling Technology, 2118T) and horseradish peroxidase-linked anti-rabbit (1:2500, Cell Signaling Technology, 7074S) or anti-mouse secondary antibody (1:2500, Cytiva, NA931) were employed as secondary antibodies.

### 4.11 Immunofluorescence

Cells were washed three times with PBS, fixed with ice-cold 4 % paraformaldehyde containing 0.3 % Triton X-100 for 20 min at room temperature, and blocked with 5 % goat serum in PBS with 0.1 % Tween20 (PBST) for 1 h. The fixed cells were then incubated overnight at 4 °C with antibodies against EGR1 (1:500, Cell Signaling Technology, 4153), TfR1 (clone 3F3-FMA, 1:500, Sigma-Aldrich, MABC1765), MDA (clone 1E83, 1:100, JaICA, MMD-030) or COX-2 (1:400, Cell Signaling Technology, 12281) in PBS with 1 % bovine serum albumin (BSA), 5 % normal goat serum, and 0.1 % Tween20. After washing with PBST, cells were incubated with the appropriate secondary antibody for 1 h at room temperature. After washing with PBST, cells were mounted in antifade medium with DAPI. For adrenal tissue sections, double immunofluorescence labelling was performed for EGR1 and CYP11B2, EGR1 and KCNJ5, or EGR1 and MDA*.* Sections were incubated with anti-EGR1 (1:500, Cell Signaling Technology, 4153), anti-CYP11B2 (1:200, clone 17B) [1], anti-KCNJ5 (clone 36-33-5, 1:2000) [55], or anti-MDA (clone 1E83, 1:100, JaICA, MMD-030) monoclonal antibodies. Images were captured on a Leica DM2500 microscope, and imageJ was used quantification.

### 4.12 Immunohistochemistry

FFPE sections of pig and human adrenal tissues were deparaffinized, and antigen retrieval was performed by heating sections for 20 min in citrate buffer (pH = 6) or 45 min in Tris-EDTA buffer (pH = 9). Following blocking with 5 % goat serum for 1 h, sections were incubated overnight at 4 °C with monoclonal antibodies against EGR1 (1:500, Cell Signaling Technology, 4153), KCNJ5 (clone 36-33-5, 1:2000) [55], CYP11B2 (1:200, clone 17B) [1], MDA (clone 1E83, 1:200, JaICA, MMD-030), 4-HNE (1:400, Abcam, ab46545) or COX-2 (1:400, Cell Signaling Technology, 12281). After washing three times with Tris-buffered saline with Tween 20, sections were incubated with ZytoChem Plus (HRP) Polymer kit or with DAKO anti mouse/rabbit secondary antibody for 1 h. DAB solution was used for visualization. Slides were counterstained with Harris hematoxylin solution before drying and digital scanning using uSCOPE™ MXII.

### 4.13 Aldosterone assay

Aldosterone in cell culture supernatants was quantified using the chemiluminescent immunoassay method with the LIAISON® kit (DiaSorin Inc., Stillwater, MN, USA). Aldosterone concentrations were normalized to protein concentrations of total cell extracts quantified using the BCA Protein Assay Kit (Thermo Scientific, 23227).

### 4.14 Statistical analyses

Statistical analyses were conducted using SPSS software (version 25.0. IBM corp, Armonk, N.Y., US). Data normality was assessed using a Shapiro-Wilk test. For comparisons between two groups, statistical significance was determined using either the Student's t-test or the Mann-Whitney *U* test (Wilcoxon rank matched pairs test, if applicable). One-way ANOVA Dunnett-t/Bonferroni test, or a Bonferroni post-test after two-way ANOVA for comparing multiple groups. Data were presented as the mean ± standard error of mean. A *P* < 0.05 was considered statistically significant (∗*P* < 0.05, ∗∗*P* < 0.01, ∗∗∗*P* < 0.001. ∗∗∗∗*P* < 0.0001).

## CRediT authorship contribution statement

**Yingxian Pang:** Writing – original draft, Methodology, Investigation, Formal analysis, Data curation, Conceptualization. **Siyuan Gong:** Writing – review & editing, Methodology, Investigation, Formal analysis, Data curation. **Martina Tetti:** Writing – review & editing, Methodology, Investigation, Formal analysis. **Zhuolun Sun:** Writing – review & editing, Methodology, Investigation, Formal analysis, Data curation. **Sanas Mir-Bashiri:** Writing – review & editing, Methodology, Investigation. **Martin Bidlingmaier:** Writing – review & editing, Methodology, Investigation. **Thomas Knösel:** Writing – review & editing, Methodology, Investigation. **Eckhard Wolf:** Writing – review & editing, Methodology, Investigation, Funding acquisition. **Martin Reincke:** Writing – review & editing, Supervision, Project administration, Funding acquisition. **Elisabeth Kemter:** Writing – review & editing, Methodology, Investigation, Funding acquisition, Conceptualization. **Tracy Ann Williams:** Writing – original draft, Validation, Supervision, Resources, Project administration, Funding acquisition, Data curation, Conceptualization.

## Data availability statement

The data that support the findings of this study are available from the corresponding author upon reasonable request.

## Funding

This research was supported by the 10.13039/501100001659Deutsche Forschungsgemeinschaft (10.13039/501100001659DFG) project number 444776998 to T.A. Williams (WI 5359/2-1) and M. Reincke (RE 752/31-1) and project number 314061271-TRR 205 “The Adrenal: Central Relay in Health and Disease” to M. Bidlingmaier, E. Kemter, M. Reincke, T.A. Williams and E. Wolf. The research of M. Reincke is further supported by 10.13039/501100000781European Research Council under the European Union's Horizon 2020 research and innovation programme (grant agreement No. 694913) and the Else Kröner-Fresenius Stiftung (2012_A103, 2015_A228, and 2019_A104; Else-Kröner Hyperaldosteronismus-German Conn Registry). Y. Pang and S. Gong are supported by the 10.13039/501100004543China Scholarship Council, and Z. Sun is supported by a China Scholarship Council-10.13039/501100001655Deutscher Akademischer Austauschdienst (CSC-DAAD) post-doctoral fellowship (202106380162).

## Declaration of competing interest

The authors declare that they have no known competing financial interests or personal relationships that could have appeared to influence the work reported in this paper.

## Data Availability

Data will be made available on request.

## References

[1] Gomez-Sanchez C.E. (2014). Development of monoclonal antibodies against human CYP11B1 and CYP11B2. Mol. Cell. Endocrinol..

[2] Nakamura Y. (2014). Adrenal CYP11B1/2 expression in primary aldosteronism: immunohistochemical analysis using novel monoclonal antibodies. Mol. Cell. Endocrinol..

[3] Williams T.A. (2021). International histopathology consensus for unilateral primary aldosteronism. J. Clin. Endocrinol. Metab..

[4] Choi M. (2011). K+ channel mutations in adrenal aldosterone-producing adenomas and hereditary hypertension. Science.

[5] Scholl U.I. (2013). Somatic and germline CACNA1D calcium channel mutations in aldosterone-producing adenomas and primary aldosteronism. Nat. Genet..

[6] Azizan E.A. (2013). Somatic mutations in ATP1A1 and CACNA1D underlie a common subtype of adrenal hypertension. Nat. Genet..

[7] Scholl U.I. (2015). Recurrent gain of function mutation in calcium channel CACNA1H causes early-onset hypertension with primary aldosteronism. Elife.

[8] Scholl U.I. (2018). CLCN2 chloride channel mutations in familial hyperaldosteronism type II. Nat. Genet..

[9] Fernandes-Rosa F.L. (2018). A gain-of-function mutation in the CLCN2 chloride channel gene causes primary aldosteronism. Nat. Genet..

[10] Rege J. (2023). Somatic SLC30A1 mutations altering zinc transporter ZnT1 cause aldosterone-producing adenomas and primary aldosteronism. Nat. Genet..

[11] Beuschlein F. (2013). Somatic mutations in ATP1A1 and ATP2B3 lead to aldosterone-producing adenomas and secondary hypertension. Nat. Genet..

[12] Fernandes-Rosa F.L. (2014). Genetic spectrum and clinical correlates of somatic mutations in aldosterone-producing adenoma. Hypertension.

[13] De Sousa K. (2020). Genetic, cellular, and molecular heterogeneity in adrenals with aldosterone-producing adenoma. Hypertension.

[14] Nanba K. (2022). Histopathology and genetic causes of primary aldosteronism in young adults. J. Clin. Endocrinol. Metab..

[15] Omata K. (2018). Cellular and genetic causes of idiopathic hyperaldosteronism. Hypertension.

[16] Williams T.A. (2022). Adrenal surgery for bilateral primary aldosteronism: an international retrospective cohort study. Lancet Diabetes Endocrinol..

[17] Meyer L.S. (2021). Single-center prospective cohort study on the histopathology, genotype, and postsurgical outcomes of patients with primary aldosteronism. Hypertension.

[18] Prasad R. (2014). Oxidative stress and adrenocortical insufficiency. J. Endocrinol..

[19] Weigand I. (2020). Active steroid hormone synthesis renders adrenocortical cells highly susceptible to type II ferroptosis induction. Cell Death Dis..

[20] Borgonovi S.M. (2023). Docosahexaenoic acid as master regulator of cellular antioxidant defenses: a systematic review. Antioxidants.

[21] Abidi P. (2008). Oxidative stress-induced inhibition of adrenal steroidogenesis requires participation of p38 mitogen-activated protein kinase signaling pathway. J. Endocrinol..

[22] Yang W.S. (2016). Ferroptosis: death by lipid peroxidation. Trends Cell Biol..

[23] Belavgeni A. (2019). Exquisite sensitivity of adrenocortical carcinomas to induction of ferroptosis. Proc. Natl. Acad. Sci. U.S.A..

[24] Yang Y. (2021). BEX1 is differentially expressed in aldosterone-producing adenomas and protects human adrenocortical cells from ferroptosis. Hypertension.

[25] Rajamohan S.B. (2012). NADPH oxidase-derived H(2)O(2) contributes to angiotensin II-induced aldosterone synthesis in human and rat adrenal cortical cells. Antioxidants Redox Signal..

[26] Tang D. (2021). Ferroptosis: molecular mechanisms and health implications. Cell Res..

[27] Yang W.S. (2014). Regulation of ferroptotic cancer cell death by GPX4. Cell.

[28] Madrigal J.L. (2003). Induction of cyclooxygenase-2 accounts for restraint stress-induced oxidative status in rat brain. Neuropsychopharmacology.

[29] Feng H. (2020). Transferrin receptor is a specific ferroptosis marker. Cell Rep..

[30] Gong S. (2021). Primary aldosteronism: metabolic reprogramming and the pathogenesis of aldosterone-producing adenomas. Cancers.

[31] Gong S. (2023). Primary aldosteronism: spatial multiomics mapping of genotype-dependent heterogeneity and tumor expansion of aldosterone-producing adenomas. Hypertension.

[32] Xu C. (2021). Discovery of a potent glutathione peroxidase 4 inhibitor as a selective ferroptosis inducer. J. Med. Chem..

[33] Murakami M. (2015). Integration of transcriptome and methylome analysis of aldosterone-producing adenomas. Eur. J. Endocrinol..

[34] Vohra T. (2020). Effect of dietary sodium modulation on pig adrenal steroidogenesis and transcriptome profiles. Hypertension.

[35] Nogueira E.F. (2009). Role of angiotensin II-induced rapid response genes in the regulation of enzymes needed for aldosterone synthesis. J. Mol. Endocrinol..

[36] Romero D.G. (2010). Angiotensin II-regulated transcription regulatory genes in adrenal steroidogenesis. Physiol. Genom..

[37] Baek S.J. (2003). Troglitazone, a peroxisome proliferator-activated receptor gamma (PPAR gamma) ligand, selectively induces the early growth response-1 gene independently of PPAR gamma. A novel mechanism for its anti-tumorigenic activity. J. Biol. Chem..

[38] Herrine S.K. (1999). Severe hepatotoxicity associated with troglitazone. Ann. Intern. Med..

[39] Pagel J.I. (2012). Disease progression mediated by egr-1 associated signaling in response to oxidative stress. Int. J. Mol. Sci..

[40] Zheng S.K. (2024). Oxidative stress-induced EGR1 upregulation promotes NR4A3-mediated nucleus pulposus cells apoptosis in intervertebral disc degeneration. Aging (Albany NY).

[41] Cheff D.M. (2023). The ferroptosis inducing compounds RSL3 and ML162 are not direct inhibitors of GPX4 but of TXNRD1. Redox Biol..

[42] Liu T. (2023). ALOX5 deficiency contributes to bladder cancer progression by mediating ferroptosis escape. Cell Death Dis..

[43] Lin Z. (2024). EGR1 promotes erastin-induced ferroptosis through activating nrf2-HMOX1 signaling pathway in breast cancer cells. J. Cancer.

[44] Fan K. (2021). The Egr-1/miR-15a-5p/GPX4 axis regulates ferroptosis in acute myocardial infarction. Eur. J. Pharmacol..

[45] Wang K. (2024). Reactivation of MAPK-SOX2 pathway confers ferroptosis sensitivity in KRAS(G12C) inhibitor resistant tumors. Redox Biol..

[46] Xie Y. (2017). The tumor suppressor p53 limits ferroptosis by blocking DPP4 activity. Cell Rep..

[47] Sousa K.M. (2007). Microarray analyses support a role for Nurr1 in resistance to oxidative stress and neuronal differentiation in neural stem cells. Stem Cell..

[48] Yu S. (2022). BMS-470539 attenuates oxidative stress and neuronal apoptosis via mc1r/cAMP/PKA/Nurr1 signaling pathway in a neonatal hypoxic-ischemic rat model. Oxid. Med. Cell. Longev..

[49] Nogueira E.F. (2010). Regulation of aldosterone synthase by activator transcription factor/cAMP response element-binding protein family members. Endocrinology.

[50] Murakami M. (2024). Single-Nucleus analysis reveals tumor heterogeneity of aldosterone-producing adenoma. Hypertension.

[51] Yang Z. (2023). ACTL6A protects gastric cancer cells against ferroptosis through induction of glutathione synthesis. Nat. Commun..

[52] Chen D.G. (2017). Inhibition of EGR1 inhibits glioma proliferation by targeting CCND1 promoter. J. Exp. Clin. Cancer Res..

[53] Santiago F.S. (1999). New DNA enzyme targeting Egr-1 mRNA inhibits vascular smooth muscle proliferation and regrowth after injury. Nat. Med..

[54] Sakakini N. (2016). A positive feed-forward loop associating EGR1 and PDGFA promotes proliferation and self-renewal in glioblastoma stem cells. J. Biol. Chem..

[55] Yang Y. (2019). Primary aldosteronism: KCNJ5 mutations and adrenocortical cell growth. Hypertension.

